# Glucokinase and glucokinase activator

**DOI:** 10.1093/lifemeta/load031

**Published:** 2023-07-13

**Authors:** Changhong Li, Yi Zhang, Li Chen, Xiaoying Li

**Affiliations:** Nanjing AscendRare and Hua Medicine, Nanjing, Jiangsu 210000, China; Hua Medicine, Shanghai 201203, China; Hua Medicine, Shanghai 201203, China; Department of Endocrinology and Metabolism, Zhongshan Hospital, Fudan University, Shanghai 200032, China

**Keywords:** glucokinases, glucokinase activator, Type 2 diabetes, pancreatic β cells, liver, glucose homeostasis

## Abstract

Glucokinase (GK) plays a pivotal role in glucose homeostasis as the glucose sensor in the pancreas and liver. Loss of function of GK results in hyperglycemia, and gain of function causes congenital hyperinsulinemic hypoglycemia. We speculate that the progressive loss of GK at both messenger RNA (mRNA) and protein levels in the islets and liver would be the key mechanism for Type 2 diabetes (T2D) pathogenesis. The development of GK activator (GKA) as an anti-diabetic drug has been endeavored for several decades. The failure of the early development of GKAs is due to the limitation of understanding the mode of GKA action. The success of dorzagliatin in the treatment of T2D has brought new hope for GK in setting a good model for repairing the underlying defects in the pancreatic islets and liver of T2D patients.

## Introduction

Glucokinase (GK or hexokinase IV) as the glucose sensor plays a pivotal role in glucose homeostasis [1]. In pancreatic β cells, it orchestrates insulin secretion in response to circulating blood glucose levels. In the liver, it facilitates glycogen storage and postprandial clearance of glucose from the bloodstream. In addition, in pancreatic α cells, it participates in the glucose-dependent regulation of glucagon secretion. Loss of function mutation of the *GCK* gene results in hyperglycemia, including maturity-onset diabetes of the young (MODY2), and gain of function mutation causes congenital hyperinsulinemic hypoglycemia. Given that insulin secretion is impaired in Type 2 diabetes (T2D), it was initially assumed that drugs that stimulate GK activity might enhance insulin secretion. Back in the 1990s, developing a GK activator (GKA) for T2D was a novel concept. The initial success by Roche demonstrated that GK pharmacological activation could serve as a novel treatment for T2D and created a gold rush in GKA discovery. However, the research on understanding the mode of action of GKAs has been limited. The failure of the early development of GKAs has forced the field to re-address basic questions about activating GK to achieve long-term benefits for T2D patients. The recent success of dorzagliatin in Chinese T2D patients has brought new hope for GK as a T2D drug target and set a good model for repairing the underlying defects in the pancreas and liver of T2D patients through a well-designed therapeutic GKA.

## GK regulates glucose homeostasis

### Regulation of insulin and glucagon secretion in the pancreas

GK is a monomeric enzyme that catalyzes the ATP-dependent conversion of glucose to glucose 6-phosphate, the first and rate-limiting step of glycolysis in the pancreas and liver. Glucose stimulating insulin secretion (GSIS) process in pancreatic β cells is linked to the glucose metabolism triggering pathway and a metabolic amplifying pathway [2]. The triggering pathway involves ATP-sensitive potassium (K_ATP_) channel-dependent depolarization, Ca^2+^ influx, and a rise in cytosolic Ca^2+^ concentrations ([Ca^2+^]), which triggers exocytosis of insulin granules. GK recognized as a glucose sensor is the first rate-limiting enzyme of glycolysis expressed in pancreatic β cells and plays a central role in the GSIS-triggering process [3]. Simultaneously, glucose activates a metabolic amplifying pathway that does not involve additional action on K_ATP_ channels or further rise in [Ca^2+^], but that augments the secretory response to the triggering Ca^2+^ signal. The amplifying pathway is active over the full period of glucose stimulation, which in the postprandial state can involve increases in circulating glucose for several hours and is estimated to account for as much as 60%–70% of insulin secreted in response to sustained glucose stimulation [4]. GK, as a key protein in insulin secretion pathways, regulates the first and second phases of insulin secretion, which is clearly illustrated by the functional impact of human genetic mutations of the *GCK* gene [5]. Mutations in *GCK* cause a spectrum of glycemic disorders. Heterozygous loss-of-function mutations of *GCK* cause mild fasting hyperglycemia irrespective of mutation severity due to compensation from the unaffected allele (MODY2). Conversely, compound heterozygous or homozygous loss-of-function mutations of *GCK* cause permanent neonatal diabetes (PNDM), with severe hyperglycemia requiring lifelong insulin treatment. In contrast, gain-of-function *GCK* mutations result in a left shift of GSIS, leading to congenital hyperinsulinism (CHI) and hypoglycemia. These *GCK* function-related genetic diseases suggest that GK function is closely related to the glucose homeostasis set point. Despite the complex genetic and environmental causes of T2D, we, therefore, speculate that the higher glucose homeostasis set point in T2D may result from impaired GK protein levels and enzymatic activities. In other words, progressive GK defects may serve as a common underlying mechanism of the pathogenesis of T2D.

**Figure 1:**
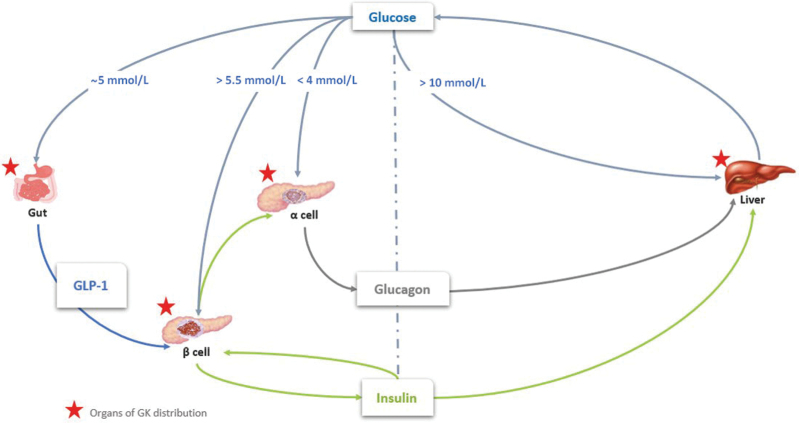
Glucokinase regulates glucose homeostasis.

The glucose homeostasis set point is mainly controlled by the glucose-dependent secretion of insulin from β cells and glucagon from α cells in a timely manner during the cycle of fasting and feeding. It has been long recognized that the thresholds for GSIS and glucose-suppressed glucagon secretion are very different. The glucose threshold for GSIS is about 5 mmol/L, while the threshold of glucose-suppressed glucagon secretion is around 3 mmol/L. It has been suggested that paracrine mechanisms play a key role in glucose suppression of glucagon secretion, i.e., insulin, zinc, γ-aminobutyric acid (GABA), etc. However, the direct role of GK in regulating glucagon release has still not been fully established. A transgenic mice study by Basco *et al*. showed that GK in pancreatic α cells regulates the glucose suppression of glucagon release [6] and generated a long debate on whether the K_ATP_ channel regulating glucagon release is GK dependent [7]. Moede *et al*. reported that GK intrinsically regulates glucose sensing and glucagon secretion in the pancreatic α cells [8]. These studies have eventually established that GK is a glucose sensor in pancreatic α and β cells and plays a central role in glucose homeostasis in the “triggering pathway” (Fig. 1).

Nevertheless, a defect of GK impacts first-phase insulin secretion and glucose-mediated intrinsic glucagon secretion in the pancreas, and thus alters the glucose set point in patients with T2D, a condition caused by low GSIS and elevated glucagon. Indeed, some studies have shown a reduction of GK expression level and activity in pancreatic β cells throughout multiple animal models and humans with obesity and diabetes. Lu *et al*. reported that impaired β-cell GK is an underlying mechanism in diet-induced diabetes [9]. High-fat diet (HFD)-induced defects in β-cell function have been linked to down-regulation of islet GK. HFD feeding reduces *GCK* mRNA and GK protein levels by up to 45% [10]. The loss of GK expression was also seen in the *ex vivo* islet study after a co-culture with palmitate. Lu *et al*. used a β cell-targeted adeno-associated virus (AAV) vector system to evaluate the HFD effect in β cells *in vitro* and *in vivo*. Increased β cell-targeted GK expression in HFD-induced diabetes restores β-cell function and improves glucose tolerance in mice, along with re-establishing the GSIS capacity of HFD islets *ex vivo*. These studies suggest that GK defects are a major underlying mechanism of T2D. Therefore, therapeutic GKA may offer a clinically differentiated approach to treat the underlying cause of diabetes by restoring the glucose homeostasis set point.

### Regulation of glucose storage and production in the liver

Liver GK is regulated at both transcriptional and posttranslational levels to ensure that its activity is inhibited when blood glucose levels are low and activated after a meal. Hepatic *GCK* expression is dependent on insulin and inhibited by glucagon. GK is also regulated at the posttranslational level by the GK regulatory protein (GCKR). GCKR binds to GK in the fasting state, which inactivates the enzyme and causes its translocation to the nucleus [11]. While glucose level is >10 mmol/L following a carbohydrate meal, GK dissociates from GCKR and translocates to the cytoplasm, where it stimulates glycolysis, glycogen synthesis, and lipogenesis. An alteration of postprandial hepatic glycogen metabolism was observed in T2D patients. A 54% reduction in net glycogen synthesis and a 30% increase in endogenous glucose production were observed in T2D patients [12]. Jiang *et al.* reported that hypermethylation of the *GCK* promoter region led to a reduction of hepatic GK expression along with a reduction of hepatic glycogen, suggesting that the reduction of hepatic GK is associated with hepatic insulin resistance [13]. Haeusler *et al*. reported a significant reduction of hepatic *GCK* gene expression level in T2D subjects with HbA1c >7% [14]. A significant decrease in pancreatic islet *GCK* gene expression was also observed in non-glucose responding T2D patients [15].

### Regulation of glucagon-like peptide-1 (GLP-1) secretion in intestinal L cells

GLP-1 is secreted from specialized intestinal neuroendocrine cells (L cells) in response to dietary nutrients, particularly carbohydrates and lipids. L cells are distributed throughout the intestine but are found in greatest numbers in the jejunum, ileum, and colon. The cells face the gut lumen, suggesting that they sense the luminal concentration of amino acids, carbohydrates, and lipids directly. Plasma levels of GLP-1 increase rapidly within just a few minutes after oral glucose in rodents and humans. Meal ingestion results in a biphasic pattern of GLP-1 secretion, with an early phase beginning within 5–15 min and a prolonged second phase following within 30–60 min [16]. With regard to glucose stimulation of GLP-1 secretion, expression of GK, the glucose sensor in cells, has been found in the mouse intestinal L cells [17]. The classical glucose-sensing machinery is mediated through glucose metabolism and the closure of K_ATP_ channels [18]. It has been well studied that in terms of the *in vivo* second phase of insulin secretion, or the “amplifying phase”, GSIS is regulated by GLP-1 and glucose-dependent insulinotropic polypeptide (GIP) gut peptides [19, 20].

Studies have shown that the Na^+^-glucose cotransporter 1 (SGLT1/SLC5A1) transporter and K_ATP_ channel-regulated GLP-1 secretion is significantly impaired in T2D patients. GLP-1 secretion quickly reaches its peak value (*C*_max_) in healthy subjects at a range of 25–30 pmol/L compared with 10–12 pmol/L for T2D subjects [21]. In an oral glucose tolerance test study of T2D patients, dorzagliatin, a GKA, alone stimulated GLP-1 secretion with an AUEC_0–4h_ increase of 103.6% and *C*_max_ increase of 141%. The *C*_max_ of total GLP-1 reached 22.1 pmol/L, the same range in normal glucose tolerance (NGT) subjects as described by Ferrannini [22, 23]. The time to reach maximum plasma concentration (*T*_max_) is 30 min for dorzagliatin-modulated GLP-1 secretion, which was similar in T2D subjects compared to NGT subjects [23, 24], suggesting that dorzagliatin acts on intestinal L cells and improves GLP-1 secretion in T2D patients. The most recent findings suggest that pancreatic α cells also secrete GLP-1 and amplify insulin secretion through GLP-1 receptors on β cells, and the intra-islet actions of GLP-1 and glucagon on both β-cell GLP-1 receptor and glucagon receptors (GcgR) are important for the paracrine interaction between α and β cells [25, 26].

## Dorzagliatin developed as a novel GKA for the anti-diabetic drug

Numerous small molecules of GKAs have been developed in the past 20 years, based on the premise that they will stimulate insulin secretion and enhance hepatic glucose uptake in diabetes [27]. These drugs activate GK by binding to its allosteric activator site and increasing the glucose affinity and/or *V*_max_ of the enzyme. However, there might be some potential risks for GK activation, which include hypoglycemia due to an excess of insulin release at low glucose level and hepatic fat accumulation due to an enhanced lipid synthesis in the liver.

GK activity is regulated by glucose. When glucose binds to GK, it changes GK’s conformation from inactive to active conformation. This binding induces an allosteric site, which is separated from the glucose binding site, for GKA. The allosteric binding of GKA to GK stabilizes the active conformation of GK and restores the GK activity. This binding mode is critical to maintaining the glucose sensor function of GK and improves the GSIS threshold from the disease state to the NGT state [9].

Despite an initial failure with GKAs in Phase 2 clinical trials, such as piragliatin and MK-0941, it has promoted the understanding of the mode of GKA action. The failure was due to clinical hypoglycemia induced by the disruption of the GSIS threshold. Allosteric GKAs developed for diabetes therapeutics can change the kinetic parameters of the GK, such as the Hill coefficient, the changes of which can result in the alteration of its glucose-­dependent activity. Clinical study of dorzagliatin was initiated in 2013, whereas, clinical development of MK-0941 has terminated due to a high incidence of hypoglycemia at the onset of the study (a 14-week dose titration) as well as a loss of efficacy in T2D patients at week 16 [28]. The high incidence of hypoglycemia of MK-0941 was due to the disruption of the GSIS threshold. MK-0941 changed the GSIS threshold to 45 mg/dL of glucose, which is similar to the value of the effects of activating GK mutations [29, 30]. The chemical structure of dorzagliatin is markedly different from that of MK-0941, and it has shown minimum disruption of Hill coefficient values of GK [31]. Preclinical studies of dorzagliatin resulted in a desirable profile of the glucose-­dependent allosteric activation of GK. Dorzagliatin is an orally bioavailable, dual-­acting full GKA that activates pancreatic and hepatic GK in a ­glucose-dependent manner to improve glycemic control in patients with T2D. Studies in diabetic rats have found that dorzagliatin demonstrated positive effects on recovering the number of insulin-secreting β cells in the pancreas and on the GK at both mRNA and protein levels in the liver [32]. In addition to effectively reducing 24-h plasma glucose levels, dorzagliatin improved the GSIS in patients with T2D who were treated for 28 days, as indicated by a significant increase in the early phase insulin secretion index (Δ_C30_/Δ_G30_) and homeostasis model assessment 2 of β-cell function (HOMA2-β) from baseline in a Phase 1 trial [33]. Patients who were treated with dorzagliatin monotherapy of 75 mg twice a day for 12 weeks in a Phase 2 trial showed significant improvement in the glucose disposition index and reduction in insulin resistance indicated by homeostasis model assessment 2 of insulin resistance (HOMA2-IR) [34].

In two Phase 3 trials, one with dorzagliatin alone in drug-naïve patients with T2D (SEED trial) and another study of dorzagliatin in combination with metformin in patients with T2D who were unable to achieve adequate glycemic control with metformin alone (DAWN trial), it was evaluated that the efficacy and long-term safety of dorzagliatin comprising 24 weeks of double-blind treatment followed by 28 weeks of open-label treatment. The results of these two Phase 3 trials showed that dorzagliatin had a greater efficacy in lowing plasma glucose levels and also had a good safety profile during the 52-week treatment period, with no drug-related serious adverse events or severe hypoglycemia that required medical assistance [35, 36].

There is some evidence that excess glucose metabolism, rather than excess glucose, causes β-cell failure. One way to do this would be partial *GCK* inhibition to reduce glucose metabolism to the same level as that found normally under euglycemia conditions. However, this strategy is challenged by the evidence that genetic variations of GK with activation and inactivation mutations lead to hypoglycemia and hyperglycemia, respectively. Clinical use of *GCK* inhibitors is also challenging because their therapeutic value depends on the extent of inhibition.

It is very reasonable that new anti-diabetic medicine should also address obesity and metabolic syndromes which are considered as the common causes of diabetes. However, increasing evidence suggests that an improvement of first-phase insulin secretion as a signal of improved β-cell function contributes to a long-term clinical benefit of weight reduction and diabetes control. Therefore, if the new anti-diabetic medicine can restore the impaired β-cell function, it will directly address the underlying causes of T2D and will have long-term benefits for T2D. The RISE study group reported that it was difficult to achieve sustained improvement of β-cell function through current blood glucose-lowering therapeutics such as metformin, insulin, and GLP-1 agonist [37]. Extraordinary weight reduction together with fat reduction in the liver and pancreas did not lead to diabetes remission in the Diabetes Remission Clinical Trial (DiRECT) study unless glucose-stimulated acute insulin secretion had been restored [38]. The DiRECT suggests that restoration of β-cell function plays a key role in diabetes remission which cannot be achieved by body weight reduction alone. The clinical benefit of dorzagliatin has been further explored in a diabetes remission study (DREAM study) with a remarkable 65.2% remission rate without significant reduction of body weight in 52 weeks after the withdrawal of dorzagliatin treatment in the SEED study. This exciting study suggests that improving β-cell function by restoration of the impaired GK function via GKA can help some patients to achieve the goal of diabetes remission.

It is conceivable that restoring GK function in the pancreatic islets is important to improving GSIS in the triggering pathway and GLP-1 secretion from the intestine in the amplifying pathway. Therefore, GKA will improve β-cell function and GSIS via a direct effect on islets and an indirect effect on L cells. We speculate that the progressive loss of GK at both mRNA and protein levels in islets and the liver is the key mechanism for diabetes pathogenesis. GKA will restore GK function and glucose homeostasis. In addition, the improvement of hepatic glucose metabolism by GKA with effective postprandial glucose reduction also benefits diabetic patients. After the approval of dorzagliatin by the Chinese Food and Drug Administration, the only clinically available GKA will bring opportunities for further exploration to address the underlying cause of T2D and to test whether GKA will delay or reverse the diabetes progression, and to reduce diabetes complications.
